# Methicillin-Resistant *Staphylococcus aureus* in Meat Products, the Netherlands

**DOI:** 10.3201/eid1311.070358

**Published:** 2007-11

**Authors:** Inge H.M. van Loo, Bram M.W. Diederen, Paul H.M. Savelkoul, Joyce H.C. Woudenberg, Robert Roosendaal, Alex van Belkum, Nicole Lemmens-den Toom, Carlo Verhulst, Peter H.J. van Keulen, Jan A.J.W. Kluytmans

**Affiliations:** * St. Elisabeth Hospital, Tilburg, the Netherlands; † VUmc Medical University, Amsterdam, the Netherlands; ‡ Erasmus Medical Center, Rotterdam, the Netherlands; § Amphia Hospital, Breda, the Netherlands

**Keywords:** Staphylococcus aureus, methicillin resistance, meat products, dispatch

## Abstract

A new methicillin-resistant *Staphylococcus aureus* (MRSA) clone related to pig and cattle farming was detected in the Netherlands. We investigated the extent of *S. aureus* presence in meat and found 36 *S. aureus* strains in 79 samples. Two strains were MRSA; 1 was multilocus sequence type 398, the clone related to farming.

In 2003 a new clone of methicillin-resistant *Staphylococcus aureus* (MRSA) related to pig and cattle farming emerged in the Netherlands (*1*,*2*). A survey of pigs showed that nearly 40% carried this clone (*3*). Detecting this strain was relatively easy with pulsed-field gel electrophoresis (PFGE) since it is nontypable (NT), the method used for surveillance of MRSA at the National Reference Centre for MRSA (National Institute of Public Health and the Environment, Bilthoven, the Netherlands). Further typing of NT-MRSA showed that almost all strains belonged to 1 multilocus sequence typing cluster, ST 398 (*2*). We undertook this study to determine to what extent *S. aureus,* and more specifically, MRSA, was present in Dutch meat products.

## The Study

Samples of various meat products from pigs and cattle, obtained from local supermarkets and butcher shops, were examined for contamination with methicillin-susceptible *S. aureus* (MSSA) and MRSA. A total of 79 raw meat products (pork, n = 64; beef, n = 15) were collected from 31 different shops (butcher shops, n = 5; supermarkets, n = 26) from February through May 2006. Table 1 shows how many samples were investigated per shop. A small portion of the meat products (mean 7.9 g, SD 3.97) was plated directly onto chromogenic agar for the detection of MRSA (MRSA ID; bioMérieux, La-Balme-les-Grottes, France). All sides of the meat portion were streaked over a part of the agar plate, and from this inoculated area, the material was spread by using a sterile loop. The piece of meat was then put into 5-mL enrichment broth containing Mueller-Hinton broth and 6.5% NaCl. After 24-h incubation at 35°C, the enrichment broth was subcultured on Columbia agar plates with 5% sheep’s blood (CA), a MRSA-ID plate, and 1 mL of the enrichment broth was put into a second enrichment broth containing phenol-red mannitol broth with ceftizoxime (5 μg/mL) and aztreonam (7.5 μg/mL) (Regional Public Health Laboratory, Groningen, the Netherlands). The second enrichment broth was subcultured on CA and MRSA-ID. All plates were incubated for 48 h at 35°C. Presumptive *S. aureus* colonies were confirmed with a latex agglutination test (Staphaurex Plus; Murex Diagnostics Ltd, Dartford, UK), a tube coagulase test with rabbit plasma, and DNase (DNase agar; Oxoid Ltd, Basingstoke, UK). Confirmation of methicillin resistance and *S. aureus* species identification was performed by an in-house–developed, validated duplex real-time PCR for the *mecA* gene and the *S. aureus*–specific 442-bp fragment described by Martineau et al. (*4*; P.H.M. Savelkoul and A.M.C. Bergmans, pers. comm.). Susceptibility to cefoxitin and doxycycline was determined by using disk diffusion according to the Clinical Laboratory Standards Institute (formerly National Committee for Clinical Laboratory Standards) standards (*5*). All isolated *S. aureus* strains (MSSA and MRSA) were genotyped by amplified fragment gel electrophoresis (AFLP) (*6*). *Spa* types were defined according to the procedure previously described by Harmsen et al. (*7*).

**Table 1:** *Staphylococcus aureus* in meat samples, the Netherlands, 2006

No. samples/shop	No. shops	No. *S. aureus–*positive samples
1	14	7
2	6	3
3	4	3
4	3	3
5	2	2
9	1	1
10	1	0

Direct inoculation of plates yielded no MRSA-positive isolates (Table 2). The first enrichment broth yielded *S. aureus* from 30 positive meat samples, 25 pork and 5 beef. In 1 pork sample, 2 phenotypically different *S. aureus* isolates were found. One *S. aureus* isolate in pork meat was identified as MRSA. When the double-enrichment broth culture system was added, another 6 samples were *S. aureus* positive, 1 of which contained MRSA. Combining the results of both enrichment broth culture procedures, 34 samples were positive, harboring 36 phenotypically different *S. aureus* isolates (Table 2). Twenty-seven (42.2%) pork samples and 5 (33.3%) beef samples harbored *S. aureus*. Two pork samples yielded 2 phenotypically different *S. aureus* isolates. Two isolates from pork (2.5% of total samples) were found to be methicillin resistant. A total of 19 shops (61.3%) had at least 1 positive meat sample.

**Table 2:** Number of MSSA and MRSA strains in pork and beef, by culture system, the Netherlands, 2006*†

Meat	Total no. samples	Single enrichment broth				Single and double enrichment broth		
		MSSA strains	MRSA strains	No. positive samples		MSSA strains	MRSA strains	No. positive samples
Pork	64	24	1	24		29	2	29
Beef	15	5	0	5		5	0	5
Total	79	29	1	29		34	2	34

*MSSA, methicillin-susceptible *Staphylococcus aureus*; MRSA, methicillin-resistant *S. aureus*. †No *S. aureus* was found by direct culture.

AFLP typing showed 8 genetic lineages, covering 72.2% (26/36) of the isolated strains and a smaller number of unique sporadic isolates 27.8% (10/36) (Figure). *Spa* typing showed that in 6 of these genetic lineages, 1 *spa* type was identified, and in 1 lineage, 2 closely related *spa* types were identified (Figure).

**Figure:**
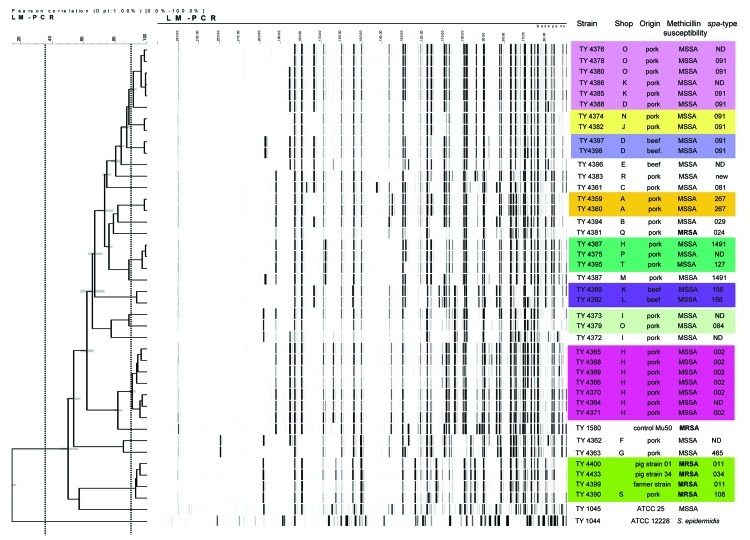
Amplified fragment gel electrophoresis typing and *spa* typing results of the *Staphylococcus aureus* isolates, methicillin susceptible (MSSA) and methicillin resistant (MRSA), in pork and beef. The boxes indicate clonally related strains. The columns indicate the strain number, the shop where the sample was bought, the origin of the sample, methicillin susceptibility, and *spa* type. ATCC, American Type Culture Collection; ND, not determined.

From the 2 samples that contained 2 phenotypically different strains, the 2 strains from 1 sample (TY4376 and TY4378) belonged to the same lineage, and the other sample contained 2 strains (TY4367 and TY4368) belonging to 2 different genetic lineages. In 5 (83.3%) of 6 shops in which >1 *S. aureus* isolate was found, typing showed clonal relationship among strains originating from the same shops (Figure).

PFGE typing of the 2 MRSA isolates showed that 1 MRSA isolate (TY4390) was nontypable by *Sma*I digestion and identical to isolates found in pigs (TY4400 and TY4433). This strain harbored *spa* type 108, which resembled the *spa* types of the pig and farmer strains (034 and 011, respectively) (*1*–*3*). These strains belong to a separate cluster in the AFLP analysis (Figure). The other MRSA isolate was identical to the US300 clone (TY 4381) and harbored *spa* type 024.

## Conclusions

To our knowledge, this is the first survey investigating the presence of MSSA and MRSA in meat products in the Netherlands. Two meat samples (2.5%) contained MRSA. Furthermore, *S. aureus* is found regularly in low amounts in meat sold to consumers. The prevalence of *S. aureus* in meat products was found to be 4%, 22.7%, and 65% in 3 other studies performed in Egypt, Switzerland, and Japan, respectively (*8*–*10*).

Contamination of the meat products could be traced back to certain abattoirs in Switzerland and poor hygienic and sanitary conditions in Egypt (*10*,*11*). The high rate of clonal relatedness of different strains within particular shops indicates cross-contamination of the meat at some point during processing. Therefore, the strain in the sample is not necessarily indicative of the strain that was carried by the animal at the source.

This study demonstrates that MRSA has entered the food chain. As the amounts were very low, the pathogen is not likely to cause disease, especially if meat is properly prepared before consumption. However, contamination of food products may be a potential threat for the acquisition of MRSA by those who handle the food. Also, a large hospital outbreak with MRSA due to contamination of food products has been described (*11*). This occurred in a hospital ward in Erasmus Medical Center in Rotterdam, the Netherlands. In this outbreak, an immunocompromised patient was probably infected by ingestion of MRSA-contaminated food, and subsequently, severe sepsis developed and the patient died. Also, an outbreak of foodborne illness caused by MRSA has been described (*12*). However, this exotoxin-mediated disease is not dependent on the methicillin susceptibility of the causative *S. aureus* strain.

All reports of MRSA in meat products described previously dealt with MRSA of human origin that was contaminating the meat. In this report, the NT-MRSA in the meat was associated with farming and is most likely of animal origin. Although the pig-related MRSA strain was found in only 1 product and in very low amounts, this finding does show that MRSA has made its way into the food chain.
